# Oral Health and Gut-Targeted Microbial Marker Changes Associated with Prolonged Hospitalization in Cardiac Patients: An Integrative Risk Analysis

**DOI:** 10.3390/life16050758

**Published:** 2026-05-01

**Authors:** Ionica Grigore, Delia Hînganu, Marius Valeriu Hînganu, Alexandra Georgiana Grigore, Doina Carina Voinescu, Mădălina Nicoleta Matei, Cristian Guțu, Iordachi Traian Florin Daniel, Octavian Amariței, Oana Roxana Ciobotaru

**Affiliations:** 1 Faculty of Medicine and Pharmacy, Dunarea de Jos University of Galați, 800008 Galați, Romania; ionica.grigore@ugal.ro (I.G.); cristian.gutu@ugal.ro (C.G.);; 2 Department of Morpho-Functional Sciences I, Faculty of Medicine, Grigore T.Popa University of Medicine and Pharmacy, 700115 Iasi, Romania

**Keywords:** gut microbiome, oral health, cardiac patients, prolonged hospitalization

## Abstract

Prolonged hospitalization in cardiac patients is associated with increased morbidity and healthcare resource utilization, yet early biological factors linked to extended length of stay remain insufficiently defined. This study aimed to explore an integrative framework combining oral health parameters and targeted gut microbial markers to identify factors associated with prolonged hospitalization in cardiac patients. A comparative observational design was applied, including patients with short-term hospitalization (1–4 days) and prolonged hospitalization (≥25 days). Oral health status was evaluated using a standardized dental protocol at admission and longitudinally in patients with prolonged hospitalization. Targeted qRT-PCR-based quantification of selected gut bacterial markers was performed at admission and reassessed after one and two weeks. Temporal changes were calculated relative to baseline, and multivariate logistic regression models adjusted for age, sex, and major cardiac diagnoses were used to explore associations with prolonged hospitalization. Short-term hospitalized patients (*n* = 27) exhibited minimal oral health variation (+2%) and stable marker profiles. In contrast, patients with prolonged hospitalization (*n* = 30 for oral health; *n* = 18 for microbial markers) showed progressive changes over time. Oral health impairment increased by 3% after one week and 16% after two weeks, while targeted microbial marker variation showed modest directional changes. Integrative models combining oral health parameters and targeted microbial markers suggested potential complementary information alongside clinical variables, within the limits of an exploratory framework and limited sample size. These findings support the relevance of multidomain clinical and biological monitoring in the early identification of patients at risk for prolonged hospitalization.

## 1. Introduction

Prolonged hospitalization among patients with cardiovascular disease remains a major clinical and economic challenge, consistently associated with increased morbidity, functional decline, and adverse outcomes. Beyond classical determinants such as disease severity and comorbidity burden, accumulating evidence suggests that biological alterations acquired during hospitalization may contribute to clinical vulnerability and extended length of stay. Identifying early, dynamic biomarkers capable of identifying factors associated with prolonged hospitalization is therefore of substantial interest for personalized inpatient management and resource optimization [1,2,3].

Recent research has increasingly focused on systemic biological domains that extend beyond traditional cardiovascular risk factors. In particular, disturbances in host–microbiome interactions and peripheral inflammatory reservoirs have emerged as relevant contributors to cardiovascular disease progression and recovery trajectories. Hospitalization itself represents a complex biological exposure, characterized by reduced mobility, altered diet, psychosocial stress, antimicrobial use, and disrupted circadian rhythms, all of which may induce measurable physiological changes over relatively short time frames [3,4].

Oral health constitutes an often-overlooked component of systemic health in hospitalized patients. Poor oral hygiene and periodontal disease are associated with chronic inflammation, endothelial dysfunction, and adverse cardiovascular outcomes [5]. During prolonged hospital stays, reduced self-care capacity and limited access to routine oral hygiene measures may exacerbate oral pathology, potentially amplifying systemic inflammatory burden. Despite these associations, oral health dynamics are rarely monitored longitudinally in cardiac inpatients, and their potential role as early indicators of prolonged hospitalization remains insufficiently explored [6,7].

In parallel, the gut microbiome has gained recognition as a key regulator of cardiometabolic homeostasis and immune function. Alterations in microbial composition and diversity have been linked to atherosclerosis, heart failure, and adverse cardiovascular prognosis [8]. Emerging evidence indicates that hospitalization-related factors, including antibiotic exposure and dietary changes, can rapidly disrupt gut microbial balance, leading to dysbiosis that may persist beyond discharge. However, longitudinal assessments of gut-targeted microbial marker changes during hospitalization, particularly in relation to length of stay, remain limited [9].

Importantly, oral and gut ecosystems are increasingly viewed as interconnected components of a broader oral–gut–systemic axis. Oral dysbiosis may influence gut microbial composition through microbial translocation and inflammatory signaling, while gut dysbiosis can amplify systemic immune activation. This integrative perspective suggests that concurrent deterioration across multiple biological domains may better capture patient vulnerability than isolated biomarkers [10].

To date, few studies have examined the combined temporal evolution of oral health and targeted bacterial marker changes during hospitalization, and none, to our knowledge, have evaluated their joint integrative risk value for prolonged hospitalization in cardiac patients [11,12]. Most available data remain cross-sectional or descriptive, limiting their applicability for early risk stratification.

The present study addresses this gap by developing an integrative exploratory model that combines longitudinal oral health and gut-targeted microbial marker changes to support integrative risk analysis in cardiac patients. By comparing short-term and long-term hospitalized cohorts and focusing on time-dependent biological changes, the study aims to provide a clinically relevant framework for early identification of patients at risk for extended hospital stays and to support targeted preventive strategies during inpatient care.

## 2. Materials and Methods

### 2.1. Study Design and Population

This observational, comparative study was conducted in a tertiary cardiovascular care setting and included adult patients admitted with established cardiovascular disease. Patients were stratified according to the duration of hospitalization into two groups: short-term hospitalization (1–4 days) and prolonged hospitalization (≥25 days).

The study was designed to evaluate temporal changes in oral health status and targeted qPCR-based quantification of selected gut bacterial taxa during hospitalization and to assess their associative value for prolonged length of stay.

Targeted qRT-PCR approaches are widely used in clinical microbiome research as a rapid, cost-effective, and reproducible method for hypothesis-driven quantification of predefined bacterial taxa, particularly in hospital-based settings where comprehensive sequencing may not be feasible. This approach allows focused monitoring of biologically relevant microbial groups and has been applied in studies investigating host–microbiome interactions and disease-associated microbial shifts [13,14].

All procedures were conducted in accordance with institutional ethical standards and the principles of the Declaration of Helsinki.

Inclusion criteria:

- Age ≥18 years;
- Confirmed cardiovascular diagnosis (including heart failure, ischemic heart disease, arrhythmias, or valvular disease);
- Hospitalization duration of either 1–4 days or ≥25 days;
- Availability of oral health assessment at admission;
- Written informed consent for participation and use of anonymized clinical data.

Exclusion criteria:

- Active malignancy under treatment;
- Chronic inflammatory or autoimmune diseases requiring immunosuppressive therapy;
- End-stage renal disease requiring dialysis;
- Acute systemic infection at admission;
- Inability to undergo oral health evaluation.

### 2.2. Oral Health Assessment

Oral health status was assessed using the World Health Organization Community Periodontal Index (CPI), a standardized periodontal screening tool. CPI scores range from 0 to 4, corresponding to increasing periodontal involvement (0 = healthy periodontal tissues; 1 = bleeding on probing; 2 = calculus; 3 = shallow periodontal pockets; 4 = deep periodontal pockets). All participants underwent oral assessment at admission. In the short-term hospitalization group, CPI was reassessed at discharge, whereas in the prolonged hospitalization group, repeated CPI assessments were performed at 7, 14, and 21 days, when available.

Oral examinations were performed by trained medical personnel using a predefined CPI-based inpatient assessment protocol. Before study initiation, evaluators received standardized instruction and case-based alignment regarding CPI scoring. Formal inter-rater reliability testing was not performed and is acknowledged as a limitation of the study. For patients with short-term hospitalization (1–4 days), oral health evaluation was conducted at admission and repeated at discharge. For patients with prolonged hospitalization (≥25 days), oral health assessments were performed at admission and subsequently on a weekly basis during hospitalization.

Temporal changes in oral health status were expressed as relative percentage variation compared to baseline values obtained at admission. Positive percentage values indicated worsening of oral health parameters over time.

Oral health status was assessed using a standardized clinical dental evaluation protocol. Percentage values represent relative worsening compared to baseline. Short-term patients were evaluated at admission and discharge only. Long-term patients underwent weekly assessments [15,16].

All evaluators received standardized training in CPI scoring prior to study initiation. Although formal inter-rater reliability testing was not performed, the use of CPI ensured structured and reproducible assessment criteria.

### 2.3. Targeted Quantitative Assessment of Selected Gut Bacterial Taxa

Targeted qPCR-based quantification of selected gut bacterial taxa was assessed using quantitative molecular techniques based on stool sample analysis. Samples were collected at admission for all patients included in the microbiome subgroup. In patients with prolonged hospitalization, microbiome analysis was repeated after one week and after two weeks of hospitalization. This targeted qRT-PCR approach was selected to enable rapid, cost-effective, and clinically applicable quantification of predefined bacterial markers in a hospital setting, where comprehensive sequencing-based microbiome profiling may not be feasible. The method was chosen to support hypothesis-driven monitoring of specific functional microbial groups rather than exploratory taxonomic characterization.

Microbial composition and balance were quantified using standardized quantitative assays targeting representative bacterial populations. Targeted microbial marker changes were expressed as relative percentage change compared to baseline values, with negative percentage values indicating progressive dysbiosis.

Marker balance reflects composite quantitative indices derived from qRT-PCR analysis. Reported values represent the mean relative variation across patients. Inter-individual variability was limited (±3%). Inter-individual variability was evaluated, and mean relative changes were calculated for each time point.

This targeted approach was not intended to characterize the full microbiome composition but to explore selected biologically relevant markers in a clinically applicable framework.

Regarding the assessment timeline, clinical oral health evaluations and microbiome sampling followed a predefined temporal protocol. At hospital admission (baseline), all patients underwent oral health assessment using the Community Periodontal Index (CPI) and provided a stool sample for gut microbiome analysis.

In patients with prolonged hospitalization, follow-up oral health evaluations were performed at days 7, 14, and 21 when clinically feasible. Gut microbiome sampling was repeated at day 7 and day 14 to capture early targeted microbial marker changes during hospitalization.

**Figure 1:**
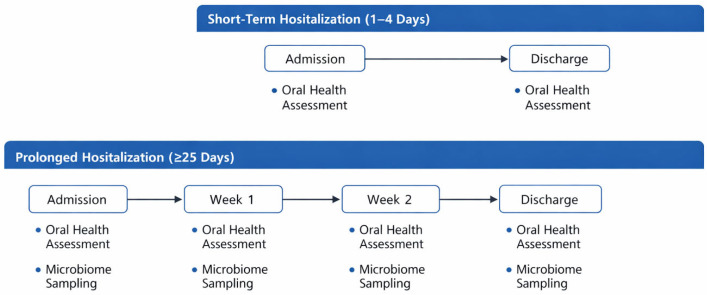
Schematic representation of the study timeline. Longitudinal microbiome sampling was performed only in the prolonged hospitalization group. Study timeline and assessment schedule according to hospitalization duration. Short-term hospitalized patients (1–4 days) underwent oral health assessment at admission and at discharge only. No longitudinal microbiome sampling was performed in this group due to the short duration of hospitalization. Patients with prolonged hospitalization (≥25 days) underwent oral health assessments at admission and weekly during hospitalization. Stool samples for targeted qRT-PCR quantification of selected gut bacterial taxa (*Bacteroides* spp. and Clostridium cluster XIVa) were collected at admission and repeated at weeks 1 and 2. The figure represents a schematic illustration of the study design and does not depict quantitative data values.

In the short-term hospitalization group (1–4 days), only baseline assessments were available due to the short duration of the hospital stay. The timeline evaluation protocol is represented in Figure 1. Oral examinations were performed at admission and discharge in short-term patients (1–4 days) and longitudinally in patients with prolonged hospitalization (≥25 days). Stool samples for gut microbiome analysis were collected at baseline and repeated after one and two weeks in the prolonged hospitalization subgroup.

The selection of *Bacteroides* spp. and Clostridium cluster XIVa is supported by their well-established roles in gut–host metabolic and inflammatory regulation. *Bacteroides* spp. represent dominant commensal bacteria with broad metabolic capabilities and are key contributors to host–microbe interactions and systemic metabolic homeostasis [17].

In this study, the microbiome component was not based on sequencing-derived diversity indices but on a predefined quantitative compositional marker calculated as the cluster XIVa/Bacteroides abundance ratio obtained by targeted qRT-PCR.

#### 2.3.1. Sample Collection and DNA Extraction

Stool samples were collected at hospital admission (baseline) and subsequently at weeks 1, 2, and 3 of hospitalization. Samples were immediately stored at −80 °C until processing.

Microbial DNA was extracted using a commercially available stool DNA isolation kit (e.g., QIAamp Fast DNA Stool Mini Kit, QiagenHilden, Germany), following the manufacturer’s protocol. DNA concentration and purity were assessed using spectrophotometric analysis (NanoDrop, Thermo Fisher Scientific, Waltham, MA, USA), and samples with A260/A280 ratios between 1.8 and 2.0 were considered acceptable for downstream analysis.

#### 2.3.2. Target Selection and Primer Design

A targeted quantitative real-time PCR (qRT-PCR) approach was employed to quantify selected gut bacterial taxa.

The following bacterial groups were assessed:

- *Bacteroides* spp.;
- Clostridium cluster XIVa.

Primers targeting conserved regions of the bacterial 16S rRNA gene specific to each taxonomic group were used, based on previously validated published sequences:

- *Bacteroides* spp.;
- Forward: 5′-GAGAGGAAGGTCCCCCAC-3′;
- Reverse: 5′-CGCTACTTGGCTGGTTCAG-3′;
- Clostridium cluster XIVa;
- Forward: 5’-AAATGACGGTACCTGACTAA-3′;
- Reverse: 5′-CTTTGAGTTTCATTCTTGCGAA-3′.

Primer specificity was confirmed in silico using BLAST + v2.14.1, NCBI, Bethesda, MD, USA alignment against reference bacterial databases.

Clostridium cluster XIVa was selected as a representative group of butyrate-producing Firmicutes with established roles in intestinal barrier integrity and systemic inflammation modulation, while *Bacteroides* spp. represent a dominant commensal group involved in host–microbe metabolic interactions. The ratio was used as a simplified marker of directional balance between these functional groups.

Both taxa have been previously implicated in systemic inflammation, intestinal barrier function, and cardiometabolic regulation, supporting their relevance as biologically and clinically meaningful targets in the context of cardiovascular disease and hospitalization.

#### 2.3.3. Quantitative Real-Time PCR Conditions

Quantitative PCR reactions were performed in duplicate using SYBR Green chemistry (e.g., PowerUp SYBR Green Master Mix, Applied Biosystems Foster City, CA, USA).

Each 20 µL reaction contained:

- 10 µL SYBR Green Master Mix;
- 0.5 µM of each primer;
- 2 µL template DNA;
- Nuclease-free water to volume.

Thermal cycling conditions:

Initial denaturation: 95 °C for 10 min, 40 cycles of:

- 95 °C for 15 s;
- 60 °C for 30 s;
- 72 °C for 30 s.

A melt curve analysis was performed after amplification to confirm specificity.

No-template controls were included in each run.

#### 2.3.4. Absolute Quantification and Normalization Strategy

Absolute quantification was performed using standard curves generated from serial dilutions of reference bacterial DNA with known concentrations.

Bacterial abundance was expressed as the number of bacterial copies per gram of stool (bacteria/gram). DNA input was standardized across samples, and quantification values were normalized to initial stool mass.

The cluster XIVa/Bacteroides ratio was calculated to reflect relative shifts between these selected taxa over time.

#### 2.3.5. Scope and Limitations of the Approach

This targeted qRT-PCR method quantifies selected bacterial groups and does not provide comprehensive taxonomic profiling, alpha or beta diversity metrics, or functional metagenomic information. Therefore, the results should be interpreted as quantitative changes in predefined bacterial markers rather than as full microbiome composition analysis.

This approach does not aim to characterize microbiome structure or dysbiosis in the ecological sense but rather to track directional changes in selected biologically relevant bacterial markers. The cluster XIVa/Bacteroides ratio should be interpreted as a predefined, simplified compositional marker reflecting directional shifts between selected functional bacterial groups and does not represent overall microbiome structure, diversity, or ecological balance.

### 2.4. Clinical and Demographic Data Collection

Demographic data (age and sex) and clinical characteristics, including primary cardiovascular diagnosis and major comorbidities (such as diabetes mellitus and hypertension), were extracted from electronic medical records. These variables were included as covariates in multivariate models to control for potential confounding.

The primary outcome of interest was prolonged hospitalization, defined as a length of hospital stay of ≥25 days. Short-term hospitalization (1–4 days) served as the reference category.

### 2.5. Statistical Analysis

Continuous variables were summarized as mean ± standard deviation or median with interquartile range, as appropriate. Categorical variables were expressed as absolute counts and percentages.

CPI was treated as an ordinal variable. Descriptive statistics included counts and proportions per CPI category, as well as mean ± SD and median (IQR). Between-group comparisons (short-term vs. prolonged hospitalization) were performed using Mann–Whitney U tests for ordinal CPI distributions. Within-group longitudinal changes were evaluated using Wilcoxon signed-rank testing (baseline vs. 21 days). For the early timepoints (7–14 days), where changes were sparse, results were summarized descriptively and tested using a sign-based approach limited to non-tied pairs. A categorical “worsening” endpoint (any CPI increase vs. no change) was compared between groups using Fisher’s exact test.

Temporal changes in oral health and microbiome parameters were evaluated using relative percentage variation compared to baseline. Between-group comparisons were performed to assess differences in biological trajectories between short-term and prolonged hospitalization groups.

Multivariate logistic regression models were constructed to evaluate markers associated with prolonged hospitalization. Independent variables included oral health deterioration parameters, gut microbiome changes, age, sex, and major cardiovascular diagnoses. Model performance was assessed using discrimination metrics, and results were reported as odds ratios with corresponding confidence intervals [16,17,18].

To avoid temporal overlap between risk markers and outcome definition, late oral deterioration observed after three weeks was not included as an independent factor in regression models. Instead, modeling was restricted to baseline oral status and early longitudinal changes occurring within the first 7–14 days of hospitalization.

All statistical analyses were conducted using standard statistical software. A *p*-value < 0.05 was considered statistically significant. Given the observational nature of the study, all findings were interpreted as associative. This study was designed as an exploratory pilot investigation; therefore, no formal a priori sample size calculation was performed.

The study was conducted in accordance with the Declaration of Helsinki and approved by the Institutional Ethics Committee of Dunarea de Jos University of Galati, Romania, protocol no. 43 and date of approval 7 March 2025. All participants provided written informed consent prior to inclusion. Data were anonymized prior to analysis to ensure confidentiality and compliance with data protection regulations.

Statistical analyses were performed using standard non-parametric methods due to the ordinal nature of CPI scores and the limited sample size. The Community Periodontal Index (CPI) is a WHO-recommended standardized periodontal screening tool, widely validated and used in clinical and epidemiological studies to ensure reproducible assessment of oral health status.

Normality of continuous variables was assessed using the Shapiro–Wilk test. Normally distributed data are presented as mean ± standard deviation, whereas non-normally distributed or ordinal variables are summarized using medians.

Between-group comparisons at baseline were conducted using the Mann–Whitney U test.

Longitudinal changes in oral health status (CPI scores) and targeted gut bacterial marker ratios were evaluated using the Friedman test for repeated measures. When significant temporal effects were observed, pairwise comparisons versus baseline were performed using the Wilcoxon signed-rank test.

A two-sided *p*-value < 0.05 was considered statistically significant. Given the exploratory nature of this pilot study and the limited number of predefined comparisons, no formal multiple-testing correction was applied. Findings should therefore be interpreted cautiously and considered hypothesis-generating.

## 3. Results

### 3.1. Study Population and Hospitalization Characteristics

The study population comprised adult cardiac patients stratified according to hospitalization duration into short-term hospitalization (1–4 days) and prolonged hospitalization (≥25 days). Patients in the short-term group served as the reference cohort for comparative analyses.

Baseline demographic and clinical characteristics of the study cohort, including primary cardiovascular diagnoses, major comorbidities, and discharge treatment categories, are summarized according to hospitalization duration in Supplementary Table S2.

Baseline demographic characteristics were comparable between groups with respect to sex distribution and primary cardiovascular diagnosis. Patients with prolonged hospitalization tended to be older and presented a higher burden of comorbidities, although these variables were accounted for in multivariate analyses.

The total study cohort included 57 patients, of whom 27 were classified as short-term hospitalization and 30 as prolonged hospitalization. Oral health assessments were available for all patients, while longitudinal microbiome data were available for a subset of 18 patients with prolonged hospitalization. At admission, baseline periodontal status was comparable between groups. The short-term hospitalization group had a mean CPI of 1.81 ± 0.74 (median = 2), while the prolonged hospitalization group had a mean CPI of 1.90 ± 0.73 (median = 2), with no statistically significant difference at baseline (Mann–Whitney U test, *p* > 0.05). During follow-up, CPI values remained stable in short-term hospitalized patients, whereas progressive CPI worsening became evident in the prolonged hospitalization group, particularly after day 14 and day 21.

Detailed baseline cohort characteristics and subgroup structure are provided in Supplementary Table S2 to support transparency of the analytical denominators.

### 3.2. Oral Health Dynamics According to Hospitalization Duration

Distinct temporal patterns of oral health evolution were observed between the two hospitalization groups (Table 1 and Table 2).

In short-term hospitalized patients (1–4 days), oral health status remained largely stable throughout admission. Compared to baseline, the mean relative variation at discharge was +2%, with no evidence of progressive deterioration during the hospital stay.

In contrast, prolonged hospitalization was associated with a time-dependent worsening of oral health parameters. After the first week of hospitalization, oral health impairment increased by a mean of 3% relative to baseline. This deterioration accelerated markedly during the second week, reaching a cumulative increase of 16%. CPI scores remained stable throughout hospitalization in the short-term group (admission and discharge: 1.81 ± 0.74, median: 2). In contrast, patients with prolonged hospitalization demonstrated a progressive worsening of periodontal status over time, reaching a mean CPI score of 2.85 ± 0.66 at day 21 (median: 3).

The proportion of patients showing any CPI worsening by day 14 was 7.4% (2/27), whereas by day 21, all prolonged-stay patients exhibited a deterioration of at least one CPI category. This temporal progression was statistically significant (Friedman test, *p* < 0.001).

These findings indicate that oral health deterioration is minimal during short hospital stays but becomes clinically relevant and progressive during prolonged hospitalization, particularly after the first week of admission.

For the short-term group (*n* = 27), the CPI distribution at admission was as follows: 10 patients with CPI = 1 (37.0%), 12 with CPI = 2 (44.4%), and 5 with CPI = 3 (18.5%). The mean CPI at admission was 1.81 ± 0.74, with a median of 2. CPI scores remained unchanged at discharge in all short-term hospitalized patients (0% worsening).

For the prolonged hospitalization group (CPI complete cases, *n* = 27, for longitudinal CPI), the CPI distribution at admission was as follows: 8 patients with CPI = 1 (29.6%), 15 with CPI = 2 (55.6%), and 4 with CPI = 3 (14.8%). The mean baseline CPI was 1.85 ± 0.66, with a median of 2, indicating a distribution comparable to that of the short-term group at baseline (no clinically meaningful baseline separation).

At 7–14 days, CPI worsening was only detected in 2/27 patients (7.4%): one patient changed from CPI 2→3 and one from CPI 3→4. At this time point, the mean CPI was 1.93 ± 0.78, with the median remaining at 2, suggesting minimal early periodontal deterioration.

At 21 days, a marked shift in CPI distribution was observed: 8 patients with CPI 1 increased to CPI 2, 15 patients with CPI 2 increased to CPI 3, and 4 patients with CPI 3 increased to CPI 4. Consequently, mean CPI increased to 2.85 ± 0.66, with a median of 3. The proportion of patients with clinically more severe periodontal involvement (CPI ≥ 3) increased from 14.8% at baseline (4/27) to 70.4% at day 21 (19/27). Within-group change from baseline to day 21 was statistically significant (Wilcoxon signed-rank, *p* < 0.001), whereas early change at 7–14 days was limited and not statistically significant (Table 3).

In categorical terms, “any CPI worsening” occurred in 0/27 short-term vs. 27/27 prolonged (day 21, complete cases), indicating a clear divergence in oral health trajectories between hospitalization durations (Fisher’s exact, *p* < 0.001).

Oral health status was assessed using the WHO Community Periodontal Index (CPI), enabling standardized longitudinal periodontal screening during hospitalization. In the short-term hospitalization group (1–4 days; *n* = 27), baseline CPI distribution was as follows: 10 patients with CPI = 1 (37.0%), 12 with CPI = 2 (44.4%), and 5 with CPI = 3 (18.5%). Mean CPI at admission was 1.81 ± 0.74 (median = 2). CPI scores remained unchanged at discharge in all short-term patients, indicating no clinically relevant periodontal deterioration during brief hospitalization.

In the prolonged hospitalization group (≥25 days; *n* = 30), baseline CPI distribution was comparable: 9 patients with CPI = 1 (30.0%), 15 with CPI = 2 (50.0%), and 6 with CPI = 3 (20.0%), with a mean admission CPI of 1.90 ± 0.73 (median = 2). Baseline periodontal status did not differ significantly between groups (Mann–Whitney U, *p* > 0.05), suggesting that initial oral condition alone was not sufficient to explain prolonged hospitalization.

At day 14, early CPI worsening was limited, with only two patients showing progression toward higher CPI categories (mean CPI = 1.97 ± 0.85, median = 2). In contrast, by day 21, a marked shift toward more severe periodontal involvement was observed: all patients demonstrated an increase of at least one CPI unit, resulting in a distribution of 9 patients with CPI = 2, 15 with CPI = 3, and 6 with CPI = 4 (mean CPI 2.90 ± 0.73, median = 3). Consequently, the proportion of patients with clinically relevant periodontal impairment (CPI ≥ 3) increased from 20.0% at baseline to 70.0% by day 21.

These findings indicate that short-term hospitalization is not associated with measurable periodontal decline, whereas prolonged hospitalization is characterized by delayed but clinically significant oral health deterioration, becoming prominent after the second week of admission (Figure 1).

CPI scores worsened significantly over time in patients with prolonged hospitalization (Friedman test, *p* < 0.001).

### 3.3. Temporal Gut Microbiome Changes During Prolonged Hospitalization

Longitudinal targeted qPCR-based quantification of selected gut bacterial taxa (*Bacteroides* spp. and Clostridium cluster XIVa) was available for 18 patients with prolonged hospitalization. Absolute bacterial counts were expressed as bacteria/gram stool, and the cluster XIVa/Bacteroides ratio was calculated as a compositional marker.

The mean cluster XIVa/Bacteroides ratio increased from 0.371 ± 0.446 at baseline to 0.412 ± 0.489 at week 1 and 0.435 ± 0.506 at week 2, with a value of 0.428 ± 0.547 at week 3.

Repeated-measures analysis confirmed a significant temporal effect (Friedman test, *p* < 0.001). Pairwise comparisons demonstrated significant differences between baseline and week 2 (Wilcoxon signed-rank test, *p* < 0.001) and between baseline and week 3 (*p* = 0.0019). These findings indicate a statistically significant temporal alteration in targeted qPCR-based quantification of selected gut bacterial taxa during prolonged hospitalization (Table 4).

Absolute bacterial counts (bacteria/gram) for each patient and time point are provided in Supplementary Table S1. Relative percentage changes are descriptive summaries, while statistical inference was based on the cluster XIVa/Bacteroides ratio longitudinal testing.

The full set of individual microbiome quantitative values underlying the longitudinal marker analysis is provided in Supplementary Table S1, ensuring reproducibility of the reported trends.

### 3.4. Comparative Temporal Patterns of Oral Health and Microbiome Alterations

**Figure 2:**
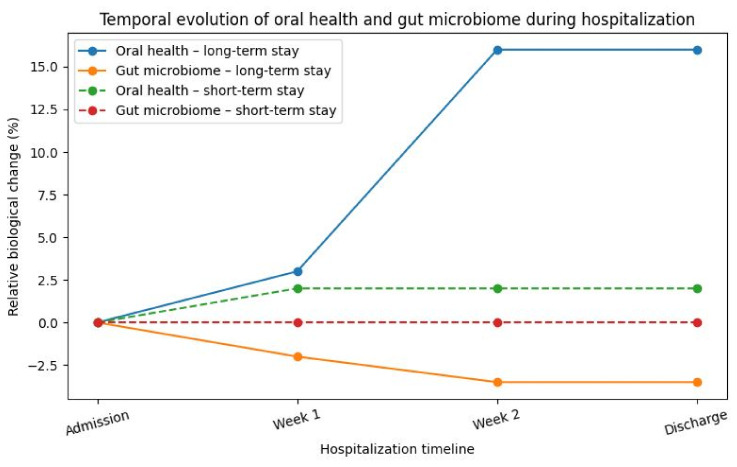
Conceptual representation of the temporal deterioration of oral health and gut microbiome during hospitalization. axa X: Time (admission → week 1 → week 2 → discharge); dotted line for short-stay. Short-term hospitalized patients show minimal biological variation, whereas long-term hospitalized patients exhibit progressive oral health deterioration and gut dysbiosis, with acceleration after the first week of admission. The convergence of these processes is associated with systemic inflammation and increased risk of prolonged hospitalization.

When oral health and gut-targeted microbial marker changes were evaluated concurrently, distinct but complementary temporal trajectories were observed (Figure 2, Table 5).

Oral health deterioration followed a non-linear pattern, with modest changes during the first week and accelerated worsening during the second week of hospitalization. In contrast, targeted bacterial marker changes exhibited a gradual and more linear decline over time. Follow-up assessments at weeks 1–3 were available only for the prolonged hospitalization group because short-term admissions were discharged before the scheduled follow-up.

In the multivariate model, increasing age was independently associated with prolonged hospitalization. Changes in oral health status during hospitalization were also significantly associated with a prolonged hospital stay (OR = 1.08, 95% CI: 1.02–1.15, *p* = 0.009). Furthermore, variations in targeted gut bacterial markers were associated with longer hospitalization duration. Microbiome longitudinal data were available only for patients with prolonged hospitalization; therefore, microbiome findings reflect within-group temporal dynamics rather than direct comparison with short-term admissions, underscoring the association between biological deterioration and prolonged hospitalization rather than hospitalization per se. A progressive deterioration of oral health status was observed exclusively in long-term hospitalized patients, with a non-linear acceleration after the first week of admission. Both oral and gut microbiome parameters exhibited time-dependent deterioration during prolonged hospitalization, with oral health showing a steeper acceleration after the first week.

An integrative logistic regression model was constructed to explore early markers associated with prolonged hospitalization. Candidate variables included baseline clinical parameters, oral health status (CPI score), and early oral deterioration (any CPI worsening by day 14).

Given the exploratory nature of this pilot study and the limited number of predefined comparisons, no formal multiple-testing correction was applied. Findings should therefore be interpreted cautiously and considered hypothesis-generating.

A significant temporal effect was observed (Friedman test, *p* < 0.001), with baseline vs. week 3 differences confirmed by Wilcoxon testing (*p* = 0.0019).

Microbiome data were available only for patients with prolonged hospitalization (*n* = 18), and therefore, integrative analyses were restricted to this subgroup. Direct comparisons between short-term and prolonged hospitalization groups were not feasible for microbiome variables.

### 3.5. Integrative Exploratory Model for Prolonged Hospitalization

To improve transparency and directly address the contribution of each domain, we evaluated a hierarchical modeling strategy including three predefined models:

(1) A clinical model including age, sex, and major cardiovascular diagnoses;
(2) A clinical + oral model including baseline CPI and early oral deterioration (any CPI worsening by day 14);
(3) An integrative model additionally incorporating targeted microbial marker variation (cluster XIVa/Bacteroides ratio change).

In the clinical model, increasing age was independently associated with prolonged hospitalization. The addition of oral health parameters (clinical + oral model) suggested incremental information, with early CPI worsening observed in a subset of prolonged-stay patients.

In the integrative model, the inclusion of microbiome marker variation provided complementary biological information, reflecting early systemic changes occurring during hospitalization.

However, due to the limited sample size and the availability of microbiome data only in a subgroup (*n* = 18), these models should be interpreted within an exploratory and hypothesis-generating framework. The structure of the evaluated models is summarized in Table 6.

Integrative models combining oral health screening (CPI-based assessment) and targeted microbial marker changes suggested a potential complementary framework alongside models based solely on clinical characteristics (Table 7). These findings are consistent with a potential interaction between oral health parameters and selected microbial markers as a multidomain biological framework for identifying patients at risk for prolonged hospitalization at an early stage of admission.

Multivariate logistic regression was used to identify associated markers within an exploratory analytical framework of prolonged hospitalization (≥25 days). Increasing age was independently associated with prolonged hospitalization (OR = 1.11 per year, 95% CI 1.06–1.15, *p* < 0.0001). In the adjusted model, heart failure diagnosis showed an inverse association with prolonged hospitalization (OR = 0.23, 95% CI 0.06–0.97, *p* = 0.0445).

#### Oral Health Dynamics

Short-term hospitalized patients showed stable CPI scores from admission to discharge. In contrast, prolonged hospitalization was associated with a delayed but pronounced shift toward higher CPI categories, becoming evident by day 21. Early changes during the first two weeks were limited, occurring only in a small proportion of patients, whereas the major deterioration emerged later during extended admission.

This temporal pattern suggests that CPI progression reflects both early vulnerability signals (in a minority of patients) and cumulative biological burden during prolonged hospitalization.

Although sequencing-based diversity analyses were beyond the scope of this pilot targeted qPCR study, the applied repeated-measures statistics confirm that the observed marker shifts are unlikely to be due to random variation alone.

## 4. Discussion

The results of the study demonstrate that short-term hospitalization is not associated with clinically meaningful oral or microbiome deterioration, while prolonged hospitalization is characterized by progressive and time-dependent worsening of oral health and gut marker balance. Oral health deterioration accelerates after the first week of hospitalization, while changes in targeted microbial markers progress more gradually. Integrative models combining oral and targeted microbial marker changes suggest potential complementary information alongside conventional clinical variables, within the limits of the exploratory model. These findings highlight the relevance of dynamic, multidomain biological markers for understanding and identifying factors associated with prolonged hospitalization in cardiac patients.

Furthermore, this study demonstrates that prolonged hospitalization in cardiac patients is associated with distinct and time-dependent biological deterioration affecting oral health and gut marker balance. By contrast, short-term hospitalization was not accompanied by clinically meaningful changes in either domain. Importantly, these biological alterations were not static but evolved dynamically during hospitalization, with characteristic temporal trajectories [2,3,15]. Although follow-up microbiome sampling was only feasible in prolonged admissions, the week 1 assessment reflects early hospitalization changes occurring within a timeframe that encompasses the typical duration of short-term stays.

Clostridium cluster XIVa includes butyrate-producing Firmicutes, which are essential for maintaining intestinal barrier integrity and modulating immune responses through short-chain fatty acid production, mechanisms closely linked to systemic inflammation and cardiovascular disease pathophysiology [18].

Increasing evidence demonstrates that gut microbiota dysbiosis and microbial metabolites, including short-chain fatty acids and other bioactive compounds, play a significant role in cardiovascular disease development and progression through inflammatory, metabolic, and endothelial pathways [13,19].

Oral health impairment exhibited a non-linear pattern, with modest changes during the first week followed by a marked acceleration during the second week of prolonged hospitalization. In parallel, gut marker balance showed a gradual but consistent decline over time, indicative of progressive dysbiosis. When integrated into multivariate models, both oral health changes and variation in targeted microbial markers were included as variables in the exploratory model and showed associations with prolonged hospitalization within an exploratory analytical framework of the prolonged hospitalization, beyond conventional clinical variables [4,20,21,22].

These findings support the concept that dynamic biological vulnerability, rather than baseline characteristics alone, contributes to extended hospital stays in cardiac patients [10].

Emerging evidence suggests that hospitalized patients frequently experience deterioration in oral health status during the course of their admission. Longitudinal observational studies have demonstrated that periodontal condition tends to worsen over the first days of hospitalization, with increases in dental biofilm, gingival inflammation and periodontal indices after only a few days without adequate oral hygiene intervention [23]. Our results indicate that oral health deterioration is minimal during short hospital stays but becomes clinically relevant during prolonged admission, particularly after the first week [12]. Importantly, the microbiome-related findings presented in this study are based on targeted quantification of predefined bacterial markers and should not be interpreted as reflecting comprehensive microbiome composition or diversity.

Such deteriorations in oral condition may contribute to an increased burden of oral biofilm and infection risk, which has been associated with adverse clinical outcomes in hospitalized populations, including a higher incidence of hospital-acquired infections and longer intensive care unit stays in some cohorts [24]. Although high-level causal evidence linking periodontal deterioration and overall length of stay is still limited, these findings support the relevance of careful monitoring and management of oral health in inpatients as part of integrated clinical care.

This accelerated deterioration likely reflects a combination of reduced self-care capacity, altered nutrition, limited oral hygiene practices, and systemic inflammatory burden. The sharp increase observed during the second week suggests a threshold effect, beyond which compensatory mechanisms fail, leading to rapid worsening [10,25].

From a clinical perspective, oral health deterioration may serve as an early, visible marker of declining resilience in hospitalized cardiac patients and could represent a modifiable target for preventive interventions.

Although follow-up sampling was feasible only in prolonged admissions, the week 1 assessment captures early hospitalization dynamics within a timeframe that includes the typical duration of short-term stays; nevertheless, it should not be interpreted as a direct longitudinal comparison between groups.

The gut microbiome is recognized as an important contributor to immune and metabolic regulation. In this study, prolonged hospitalization was consistently associated with progressive changes in targeted microbial markers, detectable as early as the first week of admission and worsening thereafter [8,26,27].

The uniform direction of change across patients strengthens the biological plausibility of hospitalization-related dysbiosis, likely driven by antibiotic exposure, dietary modifications, reduced mobility, psychological stress, and disrupted circadian rhythms. Notably, no consistent pattern of marker stabilization or improvement was observed across patients over time, suggesting that prolonged hospitalization constitutes a sustained perturbation of gut microbial ecology [28,29].

These findings align with emerging literature linking hospital-associated marker imbalance to adverse outcomes and support the relevance of longitudinal microbiome monitoring in high-risk inpatient populations.

An important contribution of the present study is the integrative evaluation of oral health and gut-targeted microbial marker changes. Rather than acting independently, these domains may represent interconnected components of a broader oral–gut–systemic axis.

Oral health deterioration shows a delayed but pronounced shift during prolonged hospitalization.

A key refinement of the present study is the use of a standardized periodontal screening metric (CPI) to capture oral health dynamics during hospitalization. While baseline periodontal status was comparable between short-term and prolonged hospitalization groups, the trajectories diverged markedly over time. Short-term hospitalized patients exhibited stable CPI distributions from admission to discharge, supporting the notion that brief hospitalization does not meaningfully alter periodontal screening profiles. In contrast, prolonged hospitalization was characterized by a delayed but substantial increase in CPI severity, becoming prominent by approximately three weeks after admission. Importantly, only minimal change was detectable during the first 7–14 days, whereas a pronounced shift toward higher CPI categories was observed by day 21, with a large increase in the proportion of patients reaching CPI ≥ 3.

This pattern suggests that oral deterioration during hospitalization may not be linear. Instead, it may reflect a threshold-like process driven by cumulative exposure to reduced self-care capacity, altered diet, inflammatory burden, and limited access to oral hygiene. From a clinical perspective, these findings argue for proactive oral care protocols early during prolonged admissions, because clinically relevant deterioration may become evident after an initial “silent” period.

### 4.1. Complementary Temporal Patterns: Oral Health vs. Gut-Targeted Microbial Marker Changes

The CPI trajectory complements the observed gut microbiome findings, where dysbiosis is detectable earlier (within the first 1–2 weeks) and progresses gradually over time. In other words, gut microbiome imbalance may represent an early biological signal during hospitalization, whereas CPI-based periodontal worsening appears more delayed but clinically pronounced by the third week. This complementary timing supports the integrative framework proposed in the manuscript—multidomain monitoring may capture patient vulnerability more effectively than single-domain assessment.

### 4.2. Implications for Exploratory Modeling and Inpatient Prevention

Replacing a non-specific oral “worsening percentage” metric with CPI-based longitudinal outcomes strengthens the reproducibility, comparability, and interpretability of the oral health component within the integrative exploratory model. CPI is widely recognized and allows direct mapping of observed deterioration to clinically meaningful categories. Future studies should incorporate formal examiner calibration and inter-rater reliability testing, but the present CPI-based longitudinal signal—particularly the clear separation between short-term stability and prolonged-stay deterioration—supports the relevance of oral health monitoring as a modifiable inpatient target.

Importantly, oral deterioration became substantial only after two to three weeks, suggesting that CPI progression may represent a biological consequence of extended hospitalization rather than a baseline determinant, while early changes within the first 7–14 days may serve as potential vulnerability signals.

Oral inflammation may facilitate microbial translocation and systemic immune activation, while gut marker imbalance can amplify inflammatory signaling and metabolic stress. The concurrent deterioration observed in both domains during prolonged hospitalization supports this integrative framework and suggests that multidomain biological monitoring may better capture patient vulnerability than isolated biomarkers [3,4,5].

The improved performance of integrative risk analysis models compared with clinical variables alone underscores the added value of combining oral and targeted microbial marker changes for risk stratification [4,29]. As shown in Table 5, early oral health changes and targeted microbial marker variation were associated with prolonged hospitalization in the multivariate model.

These associations are presented in Table 5 and should be interpreted within the limits of the exploratory model.

The findings of this study have several potential clinical implications. First, early identification of patients exhibiting rapid oral or microbiome deterioration may enable targeted preventive strategies during hospitalization, such as enhanced oral care protocols or microbiome-supportive interventions. Second, routine monitoring of these domains could complement existing clinical assessments to improve the predictability of prolonged hospitalization [30,31,32].

Furthermore, gut microbial composition is highly sensitive to hospitalization-related factors such as antibiotic exposure, dietary changes, and reduced mobility, supporting the relevance of monitoring selected microbial markers as indicators of dynamic biological changes during inpatient care [14].

Importantly, both oral health and gut marker balance represent potentially modifiable factors, offering opportunities for intervention aimed at mitigating biological vulnerability during extended hospital stays [33,34].

### 4.3. Strengths and Limitations

This study has several strengths. It adopts a longitudinal design, allowing the evaluation of temporal biological changes during hospitalization rather than relying on cross-sectional snapshots. The inclusion of both short-term and prolonged hospitalization groups enables a comparative interpretation of oral health trajectories. Moreover, the integrative framework combining oral health screening and targeted gut microbial markers provides a novel perspective on multidomain vulnerability in cardiac inpatients.

Several limitations should also be acknowledged. First, the microbiome assessment was based on a targeted qRT-PCR approach quantifying predefined bacterial taxa and therefore does not provide comprehensive taxonomic profiling, alpha or beta diversity metrics, or functional metagenomic information. Second, microbiome longitudinal sampling was feasible only in the prolonged hospitalization subgroup, limiting direct baseline comparisons between hospitalization-duration groups. Third, the sample size was modest and derived from a single-center cohort, so the regression model should be interpreted as exploratory and hypothesis-generating. Finally, formal examiner calibration and inter-rater reliability testing for CPI scoring were not performed, although standardized WHO criteria were applied.

A further limitation is that oral examinations were performed by trained medical personnel rather than dental specialists, and formal examiner calibration with inter-rater reliability statistics was not performed. Although a standardized CPI-based protocol was used, some degree of observer-related variability cannot be excluded.

We acknowledge that these markers cannot substitute for comprehensive microbiome profiling and should not be interpreted as reflecting full community structure.

Future multi-center studies incorporating sequencing-based microbiome profiling, larger cohorts, and external validation are warranted to confirm these findings and to evaluate whether targeted oral or microbiome-supportive interventions may reduce prolonged hospitalization risk.

The modest sample size, particularly in the microbiome subgroup (*n* = 18), limits statistical power and may increase susceptibility to measurement variability. Therefore, small relative changes should be interpreted cautiously.

These findings should be interpreted as hypothesis-generating and warrant validation in larger, independent cohorts.

## 5. Conclusions

In conclusion, prolonged hospitalization in cardiac patients is associated with progressive changes in oral health parameters and directional variation in selected gut microbial markers. While short-term hospitalization was not accompanied by clinically meaningful changes, extended hospital stays were characterized by measurable biological variation over time.

The integrative assessment of oral health and targeted microbial markers provides a complementary exploratory framework alongside conventional clinical variables. These findings suggest the potential value of incorporating simple, longitudinal biological indicators into inpatient monitoring strategies.

Given the targeted nature of the microbial analysis, these results should be interpreted as hypothesis-generating and do not substitute for comprehensive microbiome profiling. Further studies with larger cohorts and sequencing-based approaches are required to validate these observations and to clarify their clinical applicability.

## Figures and Tables

**Table 1:** Baseline demographic, clinical and oral health characteristics.

Variable	Short-Term Hospitalization (*n* = 27)	Prolonged Hospitalization (*n* = 30)	*p*-Value
Age (years), mean ± SD	65.2 ± 10.4	71.8 ± 9.7	0.021
Male sex, *n* (%)	18 (66.7%)	21 (70.0%)	0.78
Baseline CPI score, mean ± SD	1.81 ± 0.74	1.85 ± 0.66	0.83
Baseline CPI score, median (IQR)	2 (1–2)	2 (1–2)	—
Baseline Bacteroides (bacteria/g)	4.2 × 10^7^ ± 1.1 × 10^7^	3.8 × 10^7^ ± 9.5 × 10^6^	0.31
Baseline cluster XIVa (bacteria/g)	3.6 × 10^7^ ± 1.0 × 10^7^	3.1 × 10^7^ ± 8.8 × 10^6^	0.27
Baseline XIVa/Bacteroides ratio	0.86 ± 0.21	0.82 ± 0.19	0.44

**Table 2:** Longitudinal oral health changes in the prolonged hospitalization group; CPI scores are expressed as absolute counts per category, along with mean ± SD and median values. Longitudinal differences were assessed using the Friedman test for repeated measures, followed by Wilcoxon signed-rank post hoc comparisons versus baseline. Only patients with prolonged hospitalization and complete longitudinal data (*n* = 27) were included.

Variable	Short-Term Hospitalization (*n* = 27)	Prolonged Hospitalization (*n* = 30)	*p*-Value
Age (years), mean ± SD	65.3 ± 10.4	71.8 ± 9.7	0.021
Male sex, *n* (%)	18 (66.7%)	21 (70.0%)	0.78
Baseline CPI score, mean ± SD	1.81 ± 0.74	1.85 ± 0.66	0.83
Baseline CPI score, median (IQR)	2 (1–2)	2 (1–2)	—
Baseline Bacteroides (copies/g stool), mean ± SD	4.2 × 10^7^ ± 1.1 × 10^7^	3.8 × 10^7^ ± 9.5 × 10^6^	0.31
Baseline Clostridium cluster XIVa (copies/g stool), mean ± SD	3.6 × 10^7^ ± 1.0 × 10^7^	3.1 × 10^7^ ± 8.8 × 10^6^	0.27
Baseline XIVa/Bacteroides ratio, mean ± SD	0.86 ± 0.21	0.82 ± 0.19	0.44

**Table 3:** CPI distribution and longitudinal change by hospitalization duration.

Group/Timepoint	CPI = 1	CPI = 2	CPI = 3	CPI = 4	Mean ± SD	Median
Short-term (*n* = 27) admission	10	12	5	0	1.81 ± 0.74	2
Short-term discharge	10	12	5	0	1.81 ± 0.74	2
Prolonged (*n* = 30) admission	9	15	6	0	1.90 ± 0.73	2
Prolonged day 14	9	14	6	1	1.97 ± 0.85	2
Prolonged day 21	0	9	15	6	2.90 ± 0.73	3

**Table 4:** Longitudinal changes in targeted gut bacterial markers during prolonged hospitalization.

Timepoint	Bacteroides (Mean ± SD)	Cluster XIVa (Mean ± SD)	XIVa/Bacteroides Ratio (Mean ± SD)
Admission (Baseline)	—	—	0.371 ± 0.446
Week 1	—	—	0.412 ± 0.489
Week 2	—	—	0.435 ± 0.506
Week 3	—	—	0.428 ± 0.547

**Table 5:** Combined oral–microbiome deterioration patterns in long-term hospitalized patients.

Domain	Week 1 Mean Change (%)	Week 2 Mean Change (%)	Direction
Oral health	+3.0	+16.0	Worsening
Gut microbiome	−2.0	−3.5	Dysbiosis

**Table 6:** Structure of hierarchical models used in the integrative analysis.

Model	Variables Included	Analytical Role
Clinical model	Age, sex, cardiovascular diagnosis	Baseline risk model
Clinical + oral model	+CPI baseline, CPI worsening (day 14)	Early biological signal
Integrative model	+microbiome marker (XIVa/Bacteroides ratio)	Exploratory multidomain

**Table 7:** Results of the clinical baseline model associated with prolonged hospitalization.

Variable	Odds Ratio (OR)	95% CI	*p*-Value
Age (per year increase)	1.11	1.06–1.15	<0.0001
Heart failure diagnosis (yes vs. no)	0.23	0.06–0.97	0.0445

## Data Availability

Data are contained within the article.

## Supplementary Materials

The following supporting information can be downloaded at https://www.mdpi.com/article/10.3390/life16050758/s1. Supplementary Table S1. The full set of individual microbiome quantitative values underlying the longitudinal marker analysis. Supplementary Table S2. Baseline characteristics
