# Supplementary material for: Oral Health and Gut-Targeted Microbial Marker Changes Associated with Prolonged Hospitalization in Cardiac Patients: An Integrative Risk Analysis
Source: Life (Basel). 2026 May 1;16(5):758. doi: 10.3390/life16050758 (PMC13209107; doi:10.3390/life16050758)
Supplement: Supplementary file 1 [file life-16-00758-s001.zip › Supplementary Methods.pdf]

Statistical Analysis workflow and test selection

S1. Statistical workflow

The statistical analysis followed a predefined workflow to ensure transparency and reproducibility:

Data inspection and preprocessing

All datasets were screened for completeness, internal consistency, and range plausibility. Missing data were documented, and analyses were restricted to complete cases for longitudinal comparisons.

Descriptive statistics

Continuous variables were summarized as mean ± standard deviation (SD) when normally distributed.

Non-normally distributed continuous variables and ordinal variables were summarized as median (interquartile range, IQR).

Categorical variables were expressed as absolute counts and percentages.

Assessment of distributional assumptions

Normality of continuous variables was assessed using the Shapiro–Wilk test. Given the ordinal nature of CPI scores and the small sample size in longitudinal microbiome analyses, non-parametric tests were selected for primary inferential comparisons.

S2. Test selection strategy

Baseline comparisons

Between-group comparisons (short-term vs prolonged hospitalization) for ordinal variables (CPI scores) were performed using the Mann–Whitney U test.

Categorical comparisons (e.g., presence of CPI worsening) were evaluated using Fisher's exact test, due to small cell counts.

Longitudinal Analyses

Within-group longitudinal changes in CPI scores and Cluster XIVa/Bacteroides ratio were analyzed using the Friedman test for repeated measures.

When significant overall temporal effects were identified, post hoc pairwise comparisons versus baseline were conducted using the Wilcoxon signed-rank test.

All tests were two-sided.

Logistic Regression Modeling

Multivariate logistic regression was used to assess predictors of prolonged hospitalization (≥25 days).

Independent variables included baseline CPI score, early CPI worsening (within 7–14 days), microbiome ratio changes, age, sex, and major cardiovascular diagnoses.

Results were reported as odds ratios (OR) with corresponding 95% confidence intervals (CI).

Late oral deterioration (e.g., day 21) was excluded from predictive modeling to avoid temporal overlap with the outcome definition.

S3. Significance threshold and multiple testing

A two-sided p-value < 0.05 was considered statistically significant.

Given the exploratory nature of this pilot study and the limited number of predefined comparisons, no formal multiple-testing correction was applied. Results are therefore interpreted as hypothesis-generating rather than confirmatory.

S4. Software

All statistical analyses were performed using standard statistical software (e.g., SPSS / R / equivalent platform), following established biomedical statistical standards.

Variable	Short-term (1–4 days)	Prolonged (≥25 days)	p-value
**Number of patients, n**	178	2	—
**Length of stay, median (IQR), days**	4 (3–4)	63 (48–78)	0.0052
**Age, mean ± SD (years)**	61.2 ± 12.2	74.5 ± 9.2	0.1228
**Male sex, n (%)**	100 (56.2%)	0 (0.0%)	0.1962
**Primary diagnosis: Heart failure, n (%)**	169 (94.9%)	2 (100.0%)	1.0000
**Primary diagnosis: Ischemic heart disease, n (%)**	2 (1.1%)	0 (0.0%)	1.0000
**Primary diagnosis: Arrhythmia, n (%)**	1 (0.6%)	0 (0.0%)	1.0000
**Primary diagnosis: Valvular heart disease, n (%)**	0 (0.0%)	0 (0.0%)	1.0000
**Primary diagnosis: Other, n (%)**	6 (3.4%)	0 (0.0%)	1.0000
**Hypertension (text-derived), n (%)**	4 (2.2%)	0 (0.0%)	1.0000
**Diabetes (text-derived), n (%)**	31 (17.4%)	1 (50.0%)	0.3248
**Beta-blocker therapy, n (%)**	86 (48.3%)	1 (50.0%)	1.0000
**ACEI/ARB therapy, n (%)**	68 (38.2%)	2 (100.0%)	0.1499
**Anticoagulant therapy, n (%)**	1 (0.6%)	0 (0.0%)	1.0000
**Antiplatelet therapy, n (%)**	61 (34.3%)	0 (0.0%)	0.5494
**Statin therapy, n (%)**	83 (46.6%)	2 (100.0%)	0.2216
**Diuretic therapy, n (%)**	56 (31.5%)	2 (100.0%)	0.1026

Supplementary Table S2. Baseline clinical characteristics according to hospitalization duration; Demographic variables, primary cardiovascular diagnosis categories, text-derived comorbidities, and major discharge treatment classes are presented for short-term (1–4 days) and prolonged (≥25 days) hospitalization groups. Continuous variables are expressed as mean ± SD or median (IQR), and categorical variables as n (%). P-values were calculated using

Mann–Whitney U test or Fisher's exact test, as appropriate. Comorbidities were identified from diagnostic free-text entries.
